# Decadal cyclical geological atmospheric emissions for a major marine seep field, offshore Coal Oil Point, Southern California

**DOI:** 10.1038/s41598-023-28067-4

**Published:** 2023-02-21

**Authors:** Ira Leifer

**Affiliations:** Bubbleology Research International, Solvang, 93463 United States

**Keywords:** Atmospheric chemistry, Geochemistry, Atmospheric chemistry

## Abstract

The greenhouse gas, methane, budget has significant uncertainty for many sources, including natural geological emissions. A major uncertainty of geological methane emissions, including onshore and offshore hydrocarbon seepage from subsurface hydrocarbon reservoirs is the gas emissions’ temporal variability. Current atmospheric methane budget models assume seepage is constant; nevertheless, available data and seepage conceptual models suggest gas seepage can vary considerably on timescales from second to century. The assumption of steady-seepage is used because long-term datasets to characterize these variabilities are lacking. A 30-year air quality dataset downwind of the Coal Oil Point seep field, offshore California found methane, CH_4_, concentrations downwind of the seep field increased from a 1995 minimum to a 2008 peak, decreasing exponentially afterward with a 10.2-year timescale (*R*^2^ = 0.91). Atmospheric emissions, *E*_*A*_, were derived by a time-resolved Gaussian plume inversion model of the concentration anomaly using observed winds and gridded sonar source location maps. *E*_*A*_ increased from 27,200 to 161,000 m^3^ day^−1^ (corresponding to 6.5–38 Gg CH_4_ year^−1^ for 91% CH_4_ content) for 1995–2009, respectively, with 15% uncertainty, then decreased exponentially from 2009 to 2015 before rising above the trend. 2015 corresponded to the cessation of oil and gas production, which affects the western seep field. *E*_*A*_ varied sinusoidally with a 26.3-year period (*R*^2^ = 0.89) that largely tracked the Pacific Decadal Oscillation (PDO), which is driven on these timescales by an 18.6-year earth-tidal cycle (27.9-year beat). A similar controlling factor may underlie both, specifically varying compressional stresses on migration pathways. This also suggests the seep atmospheric budget may exhibit multi-decadal trends.

## Introduction

Natural geological sources contribute an estimated 43–50 Tg year^-1^^1^ of the important greenhouse gas, methane, CH_4_, to greenhouse gas budgets^2^, mainly from hydrocarbon seepage. Hydrocarbon seepage is the migration of hydrocarbons from a subsurface geological reservoir to the seabed or atmosphere through faults and fractures^3–6^. This migration is driven by a reservoir overpressure relative to hydrostatic through permeable migration pathways (faults and fractures) with the amount of overpressure and the pathway resistance determining the flow^7^.

A major uncertainty of geological CH_4_ emissions is the budget of terrestrial and marine hydrocarbon seepage due to its spatial heterogeneity, wide temporal variability, and long tail (microseepage) that is challenging to measure in a statistically significant manner^8^. Current atmospheric CH_4_ budget models assume seepage is constant^2,9^; nevertheless, available data and seepage conceptual models^1,7,10–12^ suggest gas seepage can vary considerably on timescales as long as century. Thus, the assumption of steady-seepage is likely an oversimplification.

For marine seepage, the hydrocarbons rise as bubbles, oily bubbles, and droplets from the seabed to the sea surface and atmosphere^13^. The seep CH_4_ budget contribution has significant uncertainties due to heterogeneity, temporal variability^1,14,15^, and a paucity of field observations, particularly long-term datasets^12^. Most longitudinal datasets are less than a year^16–20^.

**Figure 1:**
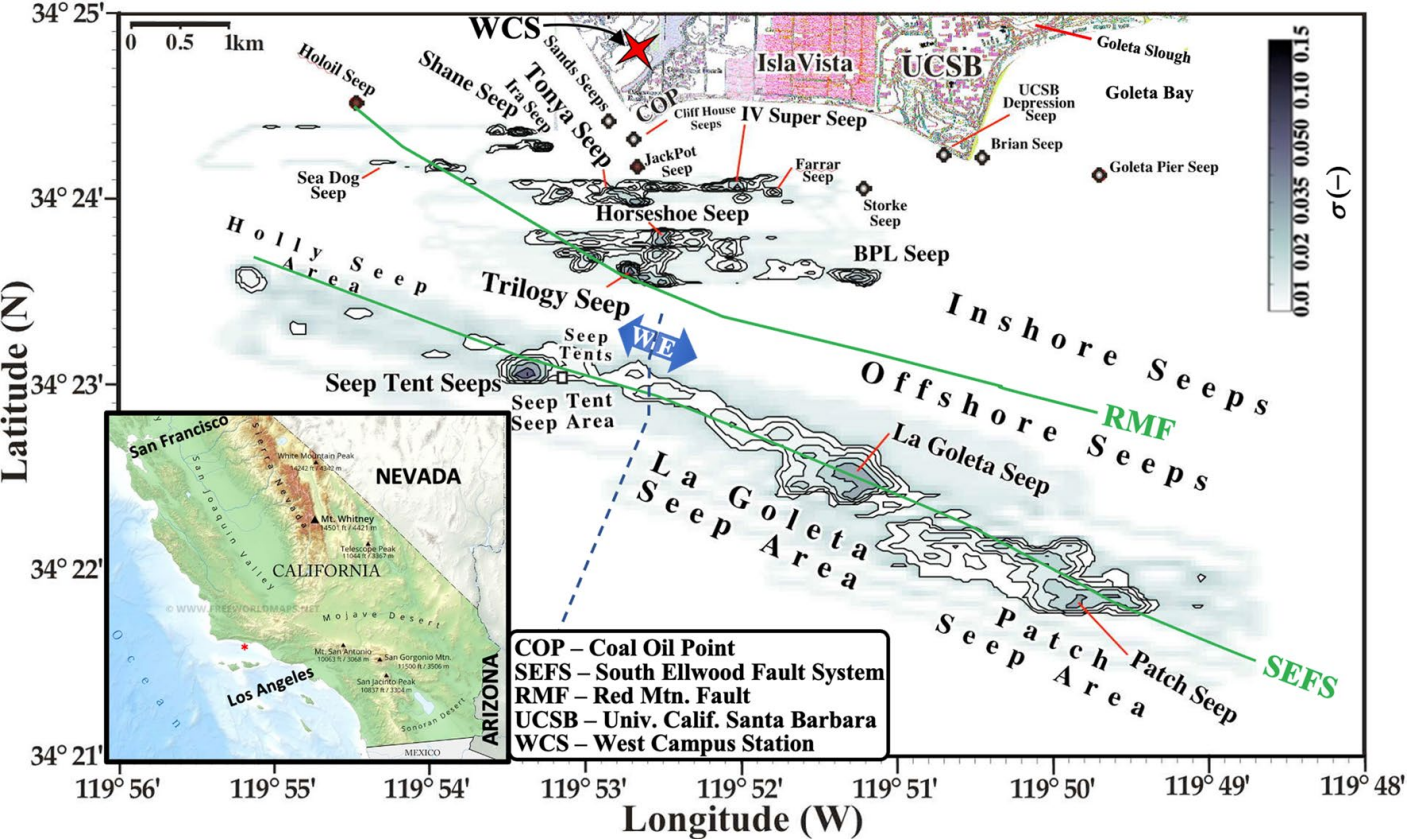
COP seep field sonar return, ω﻿, map from Leifer et al. (2010). The red star marks West Campus Station (WCS). Seep names are informal (see Supplementary Table S1), font size corresponds to strength. E-W arrow segregates east and west offshore seepage. Data keys on panels. Inset shows S. California; red star marks COP seep field. California inset map from https://www.freeworldmaps.net/united-states/california/map.html.

The Coal Oil Point (COP) seep field (Fig. 1) in the northern Santa Barbara Channel, California, is one of the largest seep fields in the world. The spatial distribution of seepage is strongly affected by geological structures^3^, which follow several trends in coastal waters extending out to a maximum water depth of ~ 85 m. Controlling geologic structures include anticlines, synclines, faults, and fault damage zones in the reservoir formation, the petroleum hydrocarbon-bearing Monterey Formation and overlying Sisquoc Formation. Seepage is non-uniformly scattered along the trends with highly active and localized seep sections associated with crossing faults and fractures^3^. Seepage in these areas decreases with distance from one or more foci, primarily along linear trends^21^.

The COP seep field lies in shallow waters from 2 to 70 m deep^12^. Seabed emissions based on sonar data were estimated at 150,000 m^3^ day^−1^ for 1994–1996^6^. Recently, Leifer et al.^22^ derived the 30-year average *E*_*B*_ at 164,000 m^3^ day^−1^ for 1990–2020 using an inversion model and a 50:50 ocean/atmosphere partition based on a study by Clark et al.^23^.

The air–water partitioning depends on the efficiency of transport across the water column, primarily bubble-mediated, as microbes largely oxidize dissolved CH_4_^24^. Bubble-mediated transport depends on seabed depth as rising bubbles lose gas to the surrounding water at a rate that decreases with rise and also depends on bubble size. Specifically, larger bubbles persist longer and thus to shallower depths, also losing less of their contents^13^. Plume strength also plays a role due to processes such as the upwelling flow—water driven upwards by the rising bubbles^25^. The upwelling flow transports dissolved gases towards the wave-mixed layer, where they can diffuse to the atmosphere^26^ with the remnant dissolved gas drifting downcurrent. The upwelling flow also decreases the time to transit the water column, enhancing bubble-mediated efficiency.

Bubble composition data at the seabed and surface demonstrate efficient vertical transport across the COP seep field water column. Seabed bubble composition is 92% CH_4_ and 7% non-methane hydrocarbons, *NMHC*, with 3–25% CO_2_. At the surface, bubble CH_4_ has decreased to 60–80% with ~ 10% *NMHC* and air gases. The remainder is primarily CO_2_, which is more soluble than CH_4_ and thus rapidly evades the bubbles, decreasing to trace levels at the sea surface^27^. The atmospheric plume of the major seep area, Trilogy Seep, found *THC* was 88.5% CH_4_^22^. Plume CO_2_ enhancement (12 ppm) was 25% that of CH_4_ (50 ppm), i.e., similar to seabed CO_2_ bubble enhancement, demonstrating efficient upwelling transport and evasion^22^. Based on a 91% CH_4_:NMHC composition, the Hornafius et al.^6^ estimate implies 36 Gg CH_4_ year^−1^. The ratio 91% ± 0.9% CH_4_:NMHC was calculated from surface sample data in Table 2 in Clark et al.^27^.

The West Campus air quality Station, WCS, is at 11-m altitude, a few kilometers from the COP seep field and half a kilometer from the coast. The terrain gently slopes down towards the coast to the southwest and towards a lagoon to the south-southeast, rising again to the bluffs at COP. These bluffs stand 11-m above sea level (Fig. 1). The WCS records *THC* concentration, *C*, wind speed, *u*, direction, *θ*, and temperature, *T*.

Seasonal and diurnal cycles in the winds and local wind patterns are important to transporting seep atmospheric emissions to WCS, where they are measured. The Santa Barbara Channel climate is Mediterranean—summer and fall are dry but generally foggy, whereas winter and spring are not foggy with infrequent storms^28^. The land/sea breeze drives diurnal wind flows–weak nocturnal offshore and stronger onshore afternoon winds^28^. Coastal mountains help maintain a shallow marine boundary layer, generally 240–300 m^28^, which typically “burns off” mid-morning. Thus, weak offshore nocturnal winds begin to veer clockwise as temperatures rise with the increase in solar insolation. Winds also strengthen. By early to mid-afternoon, typical winds are prevailing westerly (often to whitecapping), persisting into the evening before returning to the nighttime flow.

## Results

### Seep field downwind concentration trend

**Figure 2:**
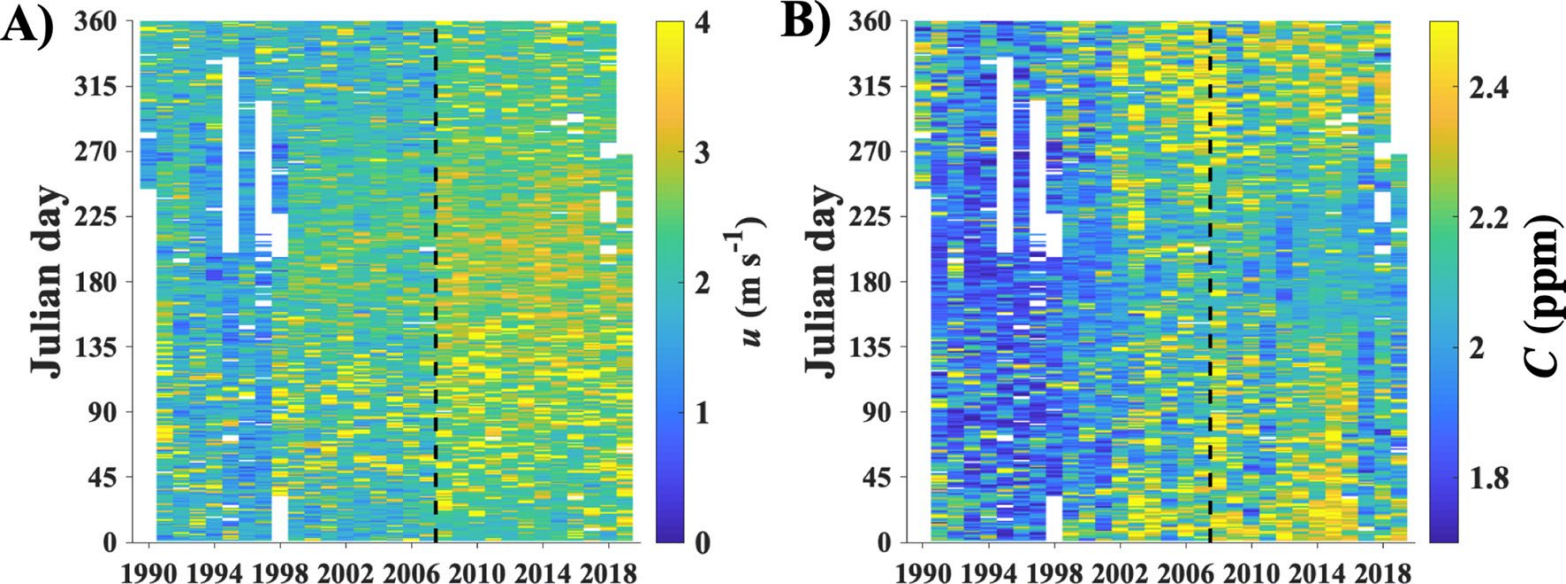
(**A**) 1-day averaged wind speed, u, and (**B**) concentration, C. Data key on panels.

**Figure 3:**
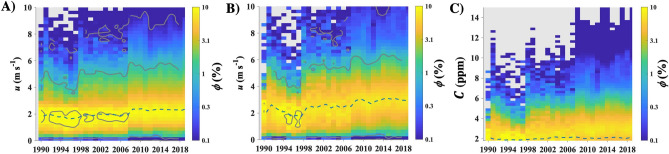
Annual wind speed, *u*, probability, *ϕ*(*u*), for (**A**) all directions, (**B**) seep directions (90° < *θ* < 270°). Contours at *ϕ* = 0.1, 1, 10%, calculated from 4-year smoothed data. (**C**) Concentration, *C*, probability, *ϕ*(*C*), for seep directions. Annual median (dashed line) shown on all panels. Data key on panels.

Significant variations on daily to seasonal to interannual time scales are apparent in the WCS time series (Supplementary Fig. S1) and the daily averages of *u* and *C* (Fig. 2). Concentrations and emissions hereafter are total hydrocarbon, *THC*, unless noted. WCS data quality improved significantly in 2008 (Fig. 1-dashed line), with measurements decreasing from 1-h to 1-min time resolution and an extended measurement range that captures higher values of *C* and *u*. A comparison of the probability distributions of *u* and *C*, *ϕ*(*u*) and *ϕ*(*C*), respectively, before and after the upgrade (Fig. 3) did not identify any significant biases^22^. WCS data 1990–2020 are available for download at^29^.

The wind speed increased gradually over the last three decades (Fig. 3), more strongly from the seep (offshore) direction, likely due to strengthening of the sea breeze circulation. Trends in *C* reflect the evolution of seep field emissions, ambient *C*, and dilution (winds). There was a gentle increase in the median of *C* from the seep field direction with a significant increase in the probability of larger values. The trend in *C* is the opposite expected trend induced by the slightly strengthening winds (Fig. 3A).

**Figure 4:**
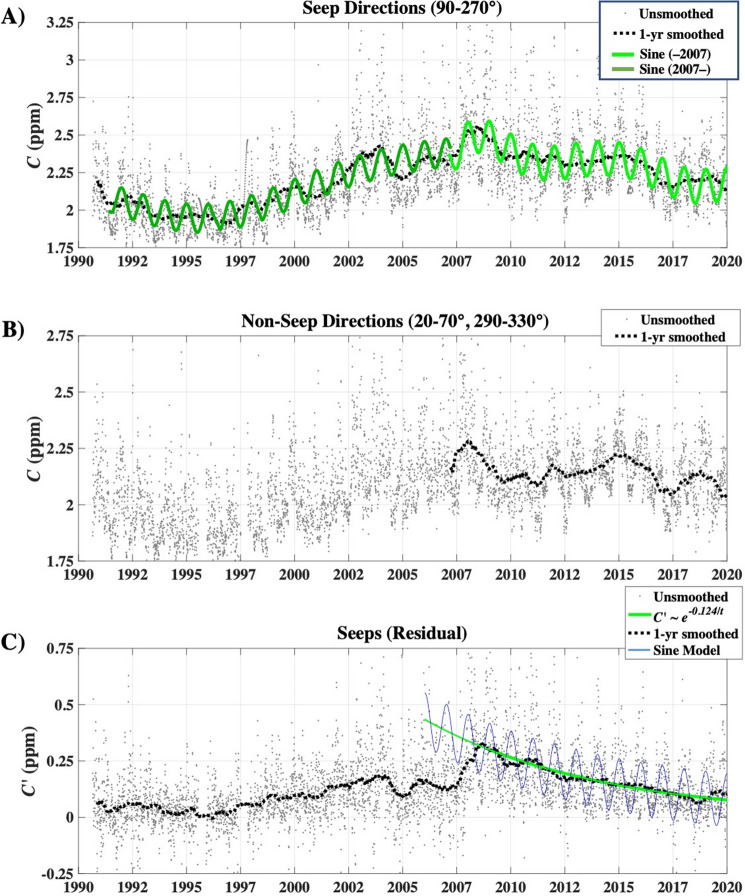
(**A**) Seep directions (90°–270°) and disjoint sinusoidal model with a shift at 2007, (**B**) channel background (260°–290°), (**C**) seep-background difference and exponential fit for 2008–2020. Data keys on panels. See supplementary Fig. S2 for $C {^{\prime}}\left(t,\theta seep\right)$ for 2008–2020.

The trend in $C\left(t,{\theta }_{seep}\right)$, where *θ*_seep_ = 90°–270°, matched the trend in seep field extent with the field waning, waxing, and then waning. Specifically, $C\left(t,{\theta }_{seep}\right)$ decreased through 1995, then increased through 2008, decreasing afterward (Fig. 4A). The seep field concentration anomaly, $C {^{\prime}}$(*t*,*θ*_seep_), was calculated by subtracting the trend of the channel background (260–290°), open Pacific in a direction without any noted seepage. $C {^{\prime}}$(*t*,*θ*_seep_) relates to seep field atmospheric emissions, *E*_*A*_, given the subtle changes in overall winds over the period (Fig. 3B). Note, the background trend includes evasion from the dissolved downcurrent plume, and also is affected by any emissions that influence overall channel CH_4_.

A two-part, 365.3-day period (124-ppb amplitude) sine function fit the seasonal trend to account with a very small phase offset around 2008 (see Supplementary Sect. S4 for seep field seasonality discussion) and winter peak. This seasonal amplitude is far larger than California CH_4_ seasonality of ~ 20 ppb from the Walnut Grove tall tower, ~ 40 ppb for the coastal Trinidad Head station in Northern California, or the 10 ppb for Mauna Loa representing the northern hemisphere (Supplementary Fig. S3B). Trinidad Head is at sea level, so unlike Walnut Grove and Mauna Loa, altitude does not play a role in the size of the seasonal cycle. Whereas the NOAA stations show a steady polynomial growth since 2007 on interannual timescales, the WCS background (Figs. 4B, S3D) shows a very different trend with much larger variations on seasonal, annual, and interannual timescales. This highlights the importance of local sources to the Santa Barbara Basin.

**Figure 5:**
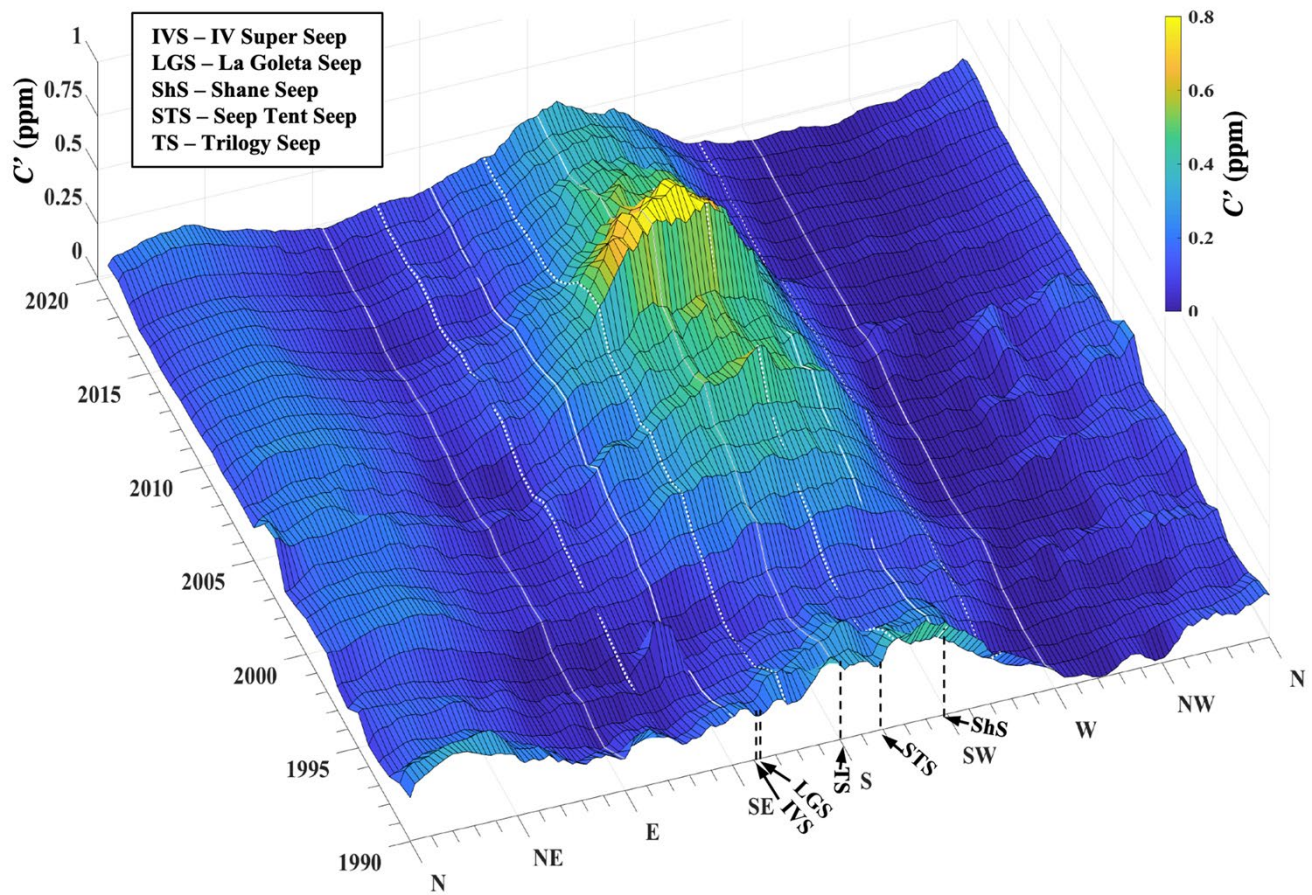
Average annualized concentration anomaly, $C{^{\prime}}\left(t,\theta \right)$, versus wind direction, *θ*, and time. Named seeps’ *θ* shown (see Supplementary Table S1); see legend for codes. See Supplementary Fig. S4B for overhead view of $C\left(t,\theta \right)$. Supplementary Fig. S4A shows an overhead view of $u\left(t,\theta \right)$.

The electrical seepage model predicts that when emissions are the low, only the highest permeability pathways remain active. The $C {^{\prime}}(t,{\theta }_{Seep}$) trend followed the field extent trend. $C{^{\prime}}(t,{\theta }_{Seep}$) decreased slowly through 1995 when emissions nearly ceased (Fig. 2B) with the remnants focused in the direction of the Seep Tent and Trilogy Seeps (Figs. 4,5). As these are two of the largest seep areas until recent years, this supports their role as the “geological” centers of the seep field for the inshore and offshore trends, respectively. From 1998 to 2004, $C{^{\prime}}(t,{\theta }_{Seep}$) increased roughly linearly before decreasing ~ 20% and then remaining roughly steady through 2007 (Fig. 2B). $C{^{\prime}}(t,{\theta }_{Seep})$ climbed to a maximum in 2009 as did seep field extent (Figs. 4, 5).

After 2008, the 1-year smoothed *C′* decreased exponentially with an 8-year half-life, *R*^2^ = 0.91 (Fig. 4C). After 2017, *C* increased slightly (Fig. 4A) while $C{^{\prime}}(t,{\theta }_{Seep})$ continues decreasing. The decreasing seep field trend for 2008–2020 is counter to the northern hemisphere and California CH_4_ trends (Supplementary Fig. S3). Note, an exponential decrease is the expected trend for a depressurizing oil and gas reservoir^30^.

### Transient seep emissions

**Figure 6:**
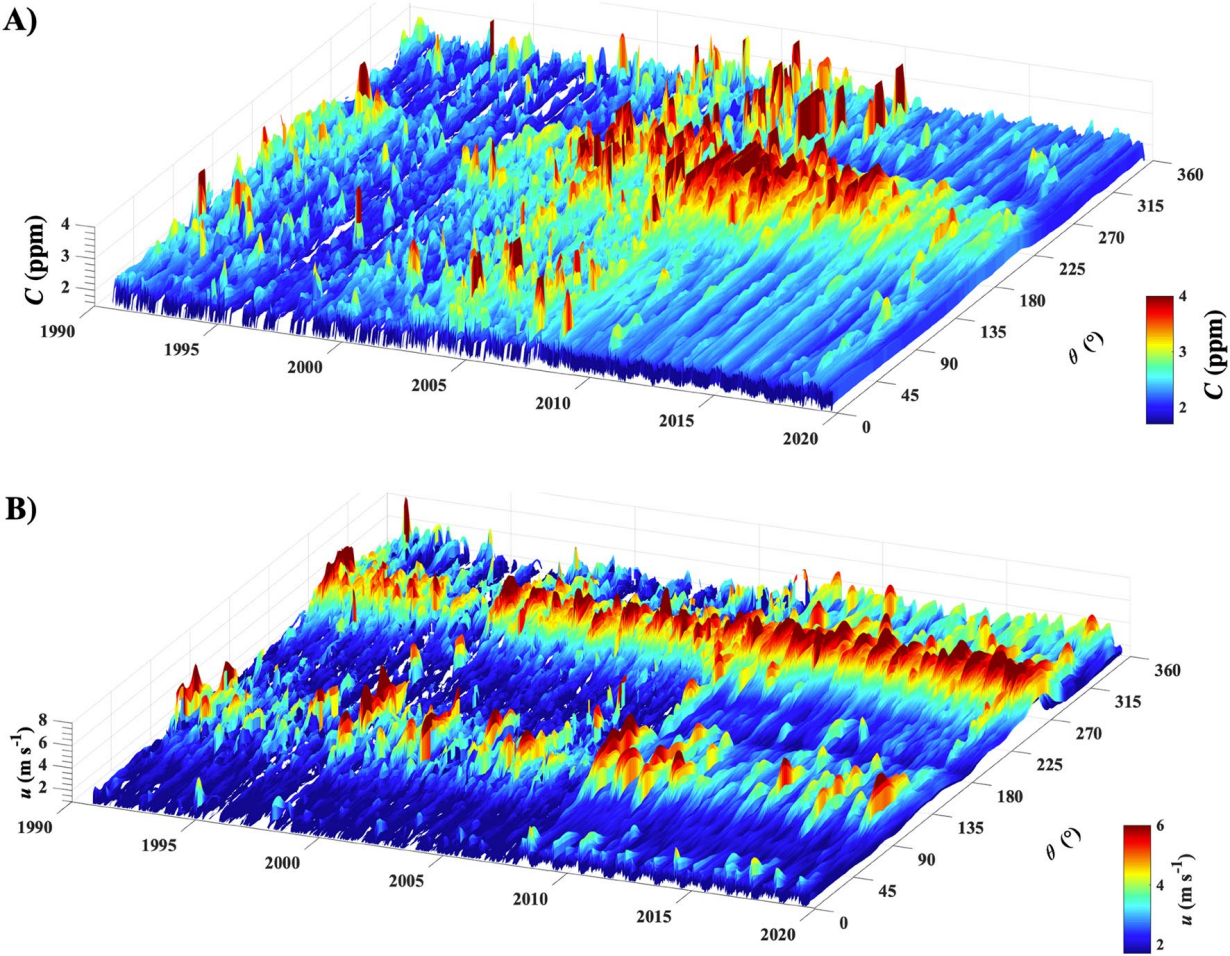
Time and direction, *θ*, resolved (**A**) average total hydrocarbon concentration, *C*_ave_(θ), and (**B**) wind speed, *u*. Data key for *C* and *u* on figure. *C* limit is clipped at 4 ppb.

Seep emissions are in two modes, continuous, albeit varying, or eruptive and transient. Transient emissions were investigated by binning *C*_seep_ with 10-day *t* bins and 10° *θ* bins with 80% overlap, yielding 2-day and 2° resolution (Fig. 6A).

During the growth period (2000–2010) and when the seep field was strong (2006–2011), eruptive emissions were more common than when emissions were weak (1992–1997) and during the 2012–2020 period of decreasing emissions (Fig. 6). Internal (geological migration) and/or external (meteorological and oceanographic) forcing factors underlie these events. For example, high wind events are accompanied by high waves that could drive the observed transient emissions, including eruptions by enabling migration breakthroughs, albeit such a mechanism is speculative. A breakthrough corresponds to a dramatic increase in permeability for an existing migration pathway or the formation of a new migration pathway^14,18,31^. Both processes are potentially driven by increased pressure in the shallow reservoir (due to deeper recharge).

Many of these events extend across the entire seep field and last for several days and even extend to directions beyond the field’s extent to the east and west. These events strongly suggest the seep field extends further northeast than mapped in recent sonar surveys. This greater extent would be consistent with a 1946 seep map (Fischer, 1978; Leifer, 2019) and suggests that generally inactive outer seepage may reactivate during eruptive events.

Stronger winds increase evasion of the downcurrent, dissolved gas plumes to the atmosphere, with feedback from higher emissions driving enhanced dissolved gas concentrations; although stronger winds also dilute plume concentrations. The dissolved plumes follow the coast—to the west-northwest under low wind conditions and eastwards under strong prevailing winds. Thus, degassing extends the *θ* range of seep field emissions. The strongest wind events tend to be either prevailing westerly or from the east-southeast. Thus, evasion from the eastwards dissolved plume (from earlier prevailing winds) is not transported towards WCS.

### Seep field emission trend

**Figure 7:**
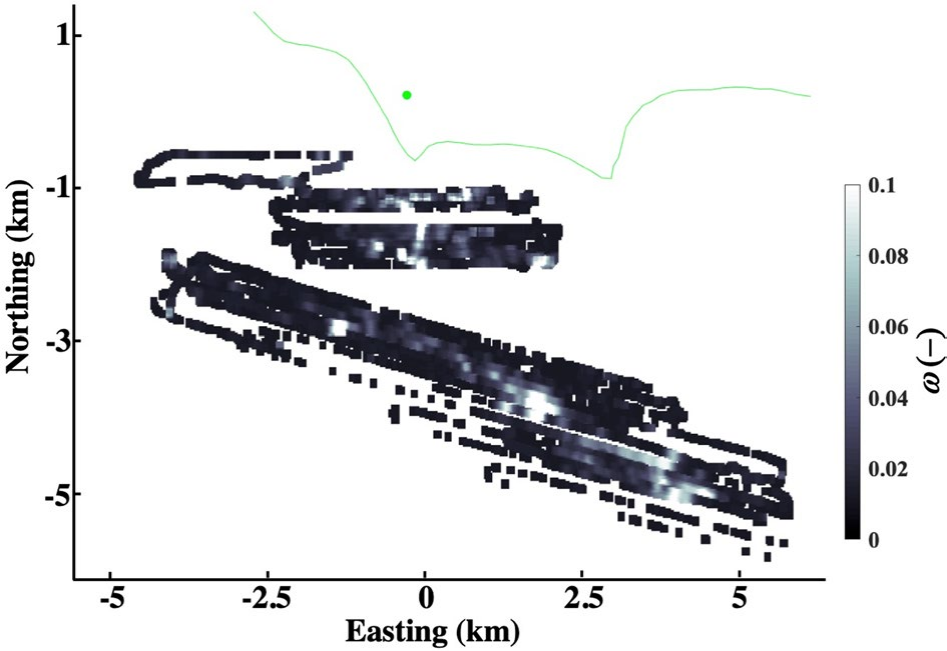
Sonar return, ω, map for 22-m grid, gap filled from 56-m gridded data. Data key on figure. Origin is at 34.414949° N, 119.879690° W.

The model calculates a Gaussian plume for each sonar grid cell above noise (Fig. 7) , which are combined to calculate $C{^{\prime}}\left(t,\theta \right)$ at WCS and is described in Leifer et al.^22^. Calculations use the observed *u*(*t*,*θ*), averaged over simulation time windows (Supplementary Fig. S4A). $C{^{\prime}}\left(t,\theta \right)$ is relative to the angular minimum in $C{^{\prime}}\left(t,\theta \right)$, which is around 270° after subtraction of a Gaussian function fit to *C*(*t*,*θ*) for the northeast 330° < *θ* < 30°^22^. This removes terrestrial emissions from the direction of suburban communities, light industry, and commercial centers.

Simulations derive the *t*-resolved atmospheric emissions, *E*_*A*_(*t*) for *dθ* = 2°, and three different time resolutions. A *Cycle* simulation used *dt* = 2-year, 0% overlap for 1990–2020, an *Annual* simulation used 1-year, 75% overlap for 2007–2020, and a *Seasonal* simulation used *dt* = 0.5-year, 50% overlap for 2007–2020. Uncertainty was ± 15% based on extensive sensitivity studies for the seep field reported in Leifer et al.^22^. Simulation uncertainty was driven primarily by uncertainty in the inshore/offshore partitioning and the boundary layer thickness. Sectoral *E*_*A*_(*t*) also were calculated for inshore seepage and offshore east and west seepage (see Fig. 1 for sector locations).

**Figure 8:**
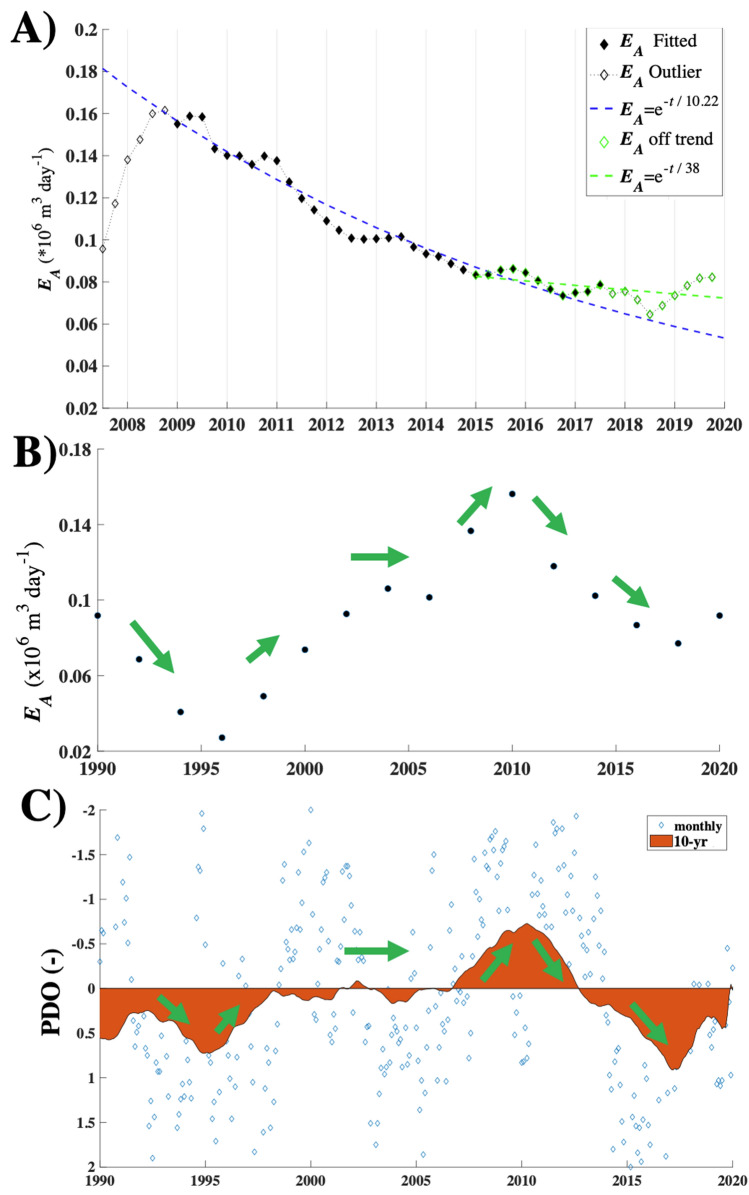
(**A**) COP seep field atmospheric emissions, *E*_*A*_, annual simulation, and least-squares linear-regression analysis exponential curve fit. (**B**) *E*_*A*_ for the Cycle simulations. See Supplementary Fig. S7–S8 for maps of *E*_*A*_ for the Cycle and Annual simulations, see text for details. (**C**) Pacific Decadal Oscillation (PDO) monthly and 10-year smoothed. Green arrows illustrate trend directions. PDO from ERDDAP^32^.

For 1990–2020 *E*_*A*_ is cyclical and is well fit (*R*^2^ = 0.89) by an offset sinusoidal function,

1 $$E_{A} \left( t \right) \, = A{\text{sin}}(t/{\text{t}} + d) \, + E_{Ac} ,$$

where A *i*s cycle amplitude (94,000 m^3^ day^−1^), τ is period (26.3-year), *E*_*Ac*_ is the cycle-average *E*_*A*_ (90,300 m^3^ day^−1^), and *d* is a fit parameter. The Cycle simulation showed similar *E*_*A*_(*t*) trends to $C{^{\prime}}(t,{\theta }_{Seep}$) with a 1995 minimum of 27,200 m^3^ day^−1^ and a 2009–2010 maximum of 156,000 m^3^ day^−1^ (*E*_*B*_ = 54,400–314,000 m^3^ day^−1^, corresponding to 13–75 Gg CH_4_ year^−1^ based on a 91% CH_4_:NMHC ratio) (Fig. 8). See Supplemental Fig. S5 for Cycle simulation with fit. The Seasonal simulation has less averaging or smoothing and found peak *E*_*A*_ = 200,000 m^3^ day^−1^ in winter 2009 (Supplementary Fig. S6). The Annual simulation *E*_*A*_(*t*) was well fit (*R*^2^ = 0.96) by an exponential for 2009–2015 with a 10.2-year timescale, after which *E*_*A*_ was above trend through 2020.

Similarity with the Pacific Decadal Oscillation (PDO) suggests emissions could be (partially) controlled by processes that underly the PDO^33^. The PDO is a long-lived El Niño-type of oscillatory trend in Pacific climate, affecting climate in across the Pacific Basin from Australia to South America to the North Pacific. The PDO is quantified by the ocean sea surface temperature anomalies in the northeast Pacific and tropical Pacific^34^.

### Seep field sector emission trends

**Figure 9:**
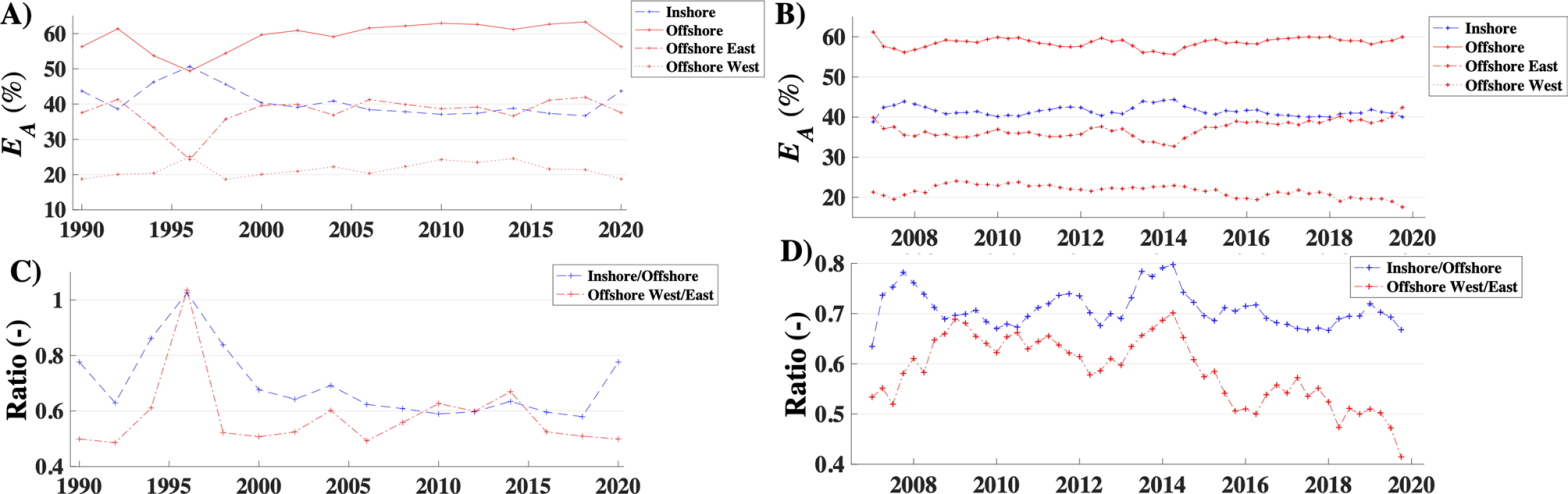
(**A**) Inshore, offshore, offshore east, and offshore west trends 1990–2020 and (**B**) 2008–2020. (**C**) Ratios of inshore to offshore and offshore east to west for 1990–2020 and (**D**) 2008–2020. 1990–2020 simulation run for 2-yr windows with 0% overlap, 22/56-m hybrid sonar grid (Fig. 7); 2007–2020 simulation run for 1-yr window with 75% overlap; 56-m sonar grid.

The model was used to understand how emissions have changed from different sectors of the seep field. Specifically, the field was segmented into inshore and offshore sectors with additional segregation of the offshore east and west sectors (Fig. 9).

Sectoral shifts were investigated to see how they related to long-term trends, with the biggest shift between the west and east offshore seep sectors. The period when emissions were at a minimum (1992–1997) corresponded to the largest relative sectoral change, with the inshore seepage growing at the expense of offshore seepage and offshore east seepage (La Goleta Seep). Meanwhile, the relative importance of the offshore and inshore sectors was largely within a narrow range from 2009 to 2020. There was a notable shift in the offshore seepage from the west to the east, which corresponded to the cessation of oil and gas production or exploitation in the west offshore sector and the above-trend rise in 2015–2016. This shift represents a diversion of hydrocarbon migration along the anticline towards the shallower crest of the offshore anticline—centered on the La Goleta Seep area. Interestingly, the Seasonal simulation shows a relative winter enhancement of inshore seepage relative to offshore seepage with winter peaks in *E*_*A*_.

## Discussion

Agreement between published sonar-derived *E*_*B*_^6,35^ and the the *E*_*A*_ in this study and is poor and significant—the disagreement significantly greater than the 15% uncertainty assessed from modeling sensitivity studies^22^. Methodological differences partially explain differences (see discussion in Supplementary Sect. S7); for example, Padilla et al.^35^ significantly under-surveyed the seep field (4.1 of 18 km^2^). However, most of the discrepancy likely arises because sonar surveys provide a snapshot of a phenomenon with high temporal variability, including episodic eruptions (Fig. 6 ), which sonar surveys likely miss. Neither sonar survey addressed seasonality—surveys are summer/fall when seas are calmest but also when seasonal emissions are comparatively low (Figs. 2, 4C). In contrast, WCS data are continuous. Thus, WCS data capture both transient and seasonal emission cycles, including seep emissions from beyond the sonar-surveyed area, providing a comprehensive characterization of emissions compared to a sonar survey.

Wind speed changes cannot account for the observed changes in *C′* and *E*_*A*_. WCS seep *C′* showed decadal-scale trends in *C′* and *E*_*A*_(*t*) with a minimum in 1995 and an unsteady increase to a peak in 2009–2010, followed by an exponential decrease. Notably, the maximum in the atmospheric emissions, *E*_*A*_, was in 2009–2010 (in both cycle and annual simulations) with a delay of 1–2 years from the peak in *C′* peak in 2008. The *E*_*A*_ cycle amplitude was 94,000 m^3^ day^−1^; or 188,000 m^3^ day^−1^ from the seabed based on a 50:50 sea-to-air partitioning^23^. This amplitude is equivalent to 45 Gg CH_4_ year^−1^ from the seabed for a 91% CH_4_ seep gas composition. No similar cycle is observed in *u* (Fig. 3).

The seep field extent waxed and waned with the emissions cycle. This pattern was consistent with the seep resistance model, wherein increasing subsurface overpressure activates new, higher migration pathways, and less favored pathways are the first to deactivate when the overpressure falls^7^. Higher seabed migration depressurizes the shallow reservoir if faster than the resupply from deeper reservoirs. Decreasing emissions (i.e., migration) allows tar deposition on migration pathways—further decreasing permeability as there is less overpressure to reopen pathways. The changes in seepage documented herein, highlight how the assumption of near steady-state seepage is poor—cycles in reservoir gas pressure build-up and release drive seep cycles^1^.

The seep field emissions and extent were minimal in 1995 (*E*_*A*_ suggests 1996 minimum), with sporadic cessation occurring in the summer of 1995 (Figs. 2, 6). 1995–1997 was a period of relatively weak winds and few storms, which increased in 1998, whereas *C′* began increasing in 1999. During this weak seepage period, emissions were centered around the Seep Tent and Trilogy Seeps.

As noted, the rate of seepage depends on the reservoir overpressure (relative to hydrostatic) and the permeability of the migration pathways. Conceptually, this can be represented by an electrical circuit where pressure corresponds to voltage, flow to current, and permeability to resistance^11,36^. Thus, factors that alter reservoir pressure alter seepage. For example, Leifer^12^ noted a dramatic increase in seepage in the vicinity of Platform Holly from the 1950s to 1970s during which platform Holly was installed and began production. Likely the drilling process fractured surrounding rock creating new migration pathways and feeding local seepage. Then, seepage decreased dramatically by the 1990s and 2000s, which was ascribed to production reducing reservoir pressure^37^. Also contributing was that geologically, seepage from the area around Platform Holly is less favored than seepage sites to the east, where the reservoir formation is shallower^12^. The shallowest portion of the offshore seepage trend underlies the La Goleta Seep, one of the major seep areas in the seep field.

Thus, the effect of the cessation of production from Platform Holly in 2015 was expected to decrease seepage, as Platform Holly production maintained the reservoir above hydrostatic. After 2008, *C′* decreased exponentially (*R*^2^ = 0.91) with an 8-year timescale through 2019—the expected decrease function of a produced oil field^30^—in this case, seepage and Platform Holly (through 2015) are “production.” An exponential decrease also was found in *E*_*A*_ for 2010 to 2015 with a longer timescale, 10.2 years. After 2015 *E*_*A*_ was above trend, consistent with Holly production withdrawals shifting to seepage.

Interestingly, the cessation of production induced shifts revealed by the sectoral analysis—specifically, a shift from the offshore west sector to the offshore east sector after 2015 (Fig. 9). This suggests that seepage was returning to its geologically preferred emissions mode at the shallowest portions of the Ellwood anticline (focused on La Goleta Seep). The implication is significant—the seepage reduction around Platform Holly 1970–1990 attributed to production^37^ was seepage that apparently was activated by the commencement of production.

Cycle asymmetry is expected as migration pathway activation requires freeing pathways of deposited tar and other sediments, leading to eruptions, whereas deactivation is the deposition that blocks migration pathways. Lower flow pathways are the first to deactivate for decreasing reservoir pressure and the last to reactivate, creating an asymmetry^7^. This asymmetry corresponds to hysteresis in an electrical model (diode) where seepage initialization requires greater voltage (overpressure) to clear a pathway than the voltage drop (lower pressure) to seal the pathway through tar deposition. As such, large transient events, e.g., as reported in Leifer et al.^31^, are predicted and observed during periods of increasing emissions (Fig. 6).

*E*_*A*_ was cyclical and well fit (*R*^2^ = 0.89) by a 26.3-year period sinusoidal (from ~ 1 cycle). The Pacific Decadal Oscillation follows a rough similarity in trends and phase—minimum in 1995, maximum in 2010, and decrease through 2017 (Fig. 8). The Pacific Decadal Oscillation is proposed to arise from several processes, including thermal anomaly advection associated with the El Nino Southern Oscillation (ENSO) modulation, and earth tides, specifically an 18.6-year period tidal cycle which has a beat at 27.9-year^33^. Earth tides can affect migration pathways by increasing and decreasing compressional stresses. Increasing compressional stress will decrease seepage by increasing resistance (decrease permeability) in migration pathways. Decreasing compressional stress induce the opposite trends. Notably, this earth tide mechanism would affect marine and terrestrial seepage on regional to large scales. This suggests that the terrestrial and marine seepage global budgets may exhibit significant multi-decadal cycles.

## Conclusion

Long-term WCS *C*_*THC*_ trends were cyclical and corresponded with seep field extent waxing and waning. This is consistent with the seep electrical model where changing resistance or reservoir pressure drives changes in seepage flow rates and due to asymmetry between seep activation/deactivation, weak seepage at the periphery (higher resistance) is the first to deactivate and the last to activate.

Time-resolved derived atmospheric emissions *E*_*A*_(*t*) showed an exponential decrease after ~ 2008 with a 10.2-year timescale that was above trend after 2015 when production in the west offshore sector ceased, which corresponded to a shift in offshore seepage to the east sector. *E*_*A*_(*t*) was cyclical with a 26.3-year period and tracked the earth tidal cycle in the Pacific Decadal Oscillation, which has an 18.6-year cycle (27.9-year beat period). This argues for a fracture compressional mechanism that increases and decreases resistance to migration that decreases and increases seepage (marine and terrestrial) emissions. The implication is that global seepage budgets may vary cyclically on multi-decade timescales.

A clear increase in seepage was demonstrated due to the cessation of production at Platform Holly, with seepage shifting back to its natural migration pathways, which had been affected by production. This suggests that as global fossil fuel production scales back with efforts to reduce anthropogenic greenhouse gas emissions and reservoir depletion to non-economic levels, the global seep emission budget likely will increase.

## Methods

### WCS data analysis

The West Campus Station, WCS, air quality station measures wind speed, *u*, and direction, *θ*, by a vane anemometer (010C,020C, Met One, Grants Pass, OR) and *THC* concentration, *C*, by a Flame Ionization Detector (51i-LT, Thermo Scientific, MA). WCS is maintained by the regulatory agency, the Santa Barbara County Air Pollution Control District. Daily instrument calibration occurs after midnight, rendering *C* unavailable 00:50–02:09 local time, LT. WCS was improved significantly in 2008 from 1-h to 1-min time resolution, which allowed far higher values of *C* and *u* due to the shorter averaging times.

WCS data began data collection in 1990. Data are hourly before 2008 and minute resolution afterward (Supplementary Fig. S1). Daily calibration is from 00:50 to 02:09 Local Time, LT. Data analysis and modeling used MATLab (MathWorks, MA). WCS data were quality controlled to remove all zero *C* values during the daily calibration and interpolating unrealistically low values (< 1.6 ppm in the 1990s and < 1.85 ppm in the 2000s). Nearest neighbor averaging smoothed the minute-resolution data after 2008.

Seasonal and longer temporal seep trends were investigated by binning $C(t,\theta )$ in time, *t*, and *θ* binned with a 30-day *t*-bin, 90% overlap, and *θ* binning of 10° bins with 80% overlap. This binning scheme smooths short-term variations while preserving monthly to interannual variations in $C(t,\theta )$. Emissions arise from numerous sources across the COP seep field; thus, different wind directions probe different portions of the seep field.

Neighbor averaging filled empty $C\left(t,\theta \right)$ *θ *bins with gaps of one or two *θ* bins. A 7-bin, 2 standard deviation running filter for *θ* identified spikes (positive and negative) in $C\left(t,\theta \right)$, which were replaced by neighbor averaging.

### Seep field emissions model

Atmospheric emissions, *E*_*A*_, are calculated by the Gaussian plume model for *u* to yield the observed *C*. The Gaussian plume model is described in Leifer et al.^22^. The model simulated $C{^{\prime}}\left(t,\theta \right)$ at WCS by initializing with gridded sonar data from Sept. 2005 with a Gaussian plume from each grid above the noise level (Fig. 7). The sonar analysis methodology is described in Leifer et al.^3^.

Specifically, the sonar return, ω, of a seep field survey collected in Sept. 2005 was hybrid gridded at 22 m in a cartesian (meter) coordinate system with origin at WCS. The approach is described in detail in Leifer et al.^22^ and Leifer et al.^3^. Gridding averages all sonar data in each bin followed by a gap-filling low-pass filter where the center bin’s value of a rolling window of 3 × 3 bins is replaced by the mean if there are more than 5 non-empty bins in the window. Additional gaps in the 22-m data are filled by replacement from a 56-m grid of the sonar data.

The sonar noise level was determined from the probability distribution of ω, specifically, where the distribution shows a shift from signal to noise domination and was 0.015. Values below the sonar noise level are set to zero.

The Gaussian plume model uses the average, time-, *t*, resolved, measured wind speed, *u*, for each wind direction, *θ*. $C{^{\prime}}\left(t\right)$ is calculated for each grid cell and added to calculate $C{^{\prime}}\left(t,\theta \right)$ at WCS. $C{^{\prime}}$(*t*,*θ*_seep_) is

1 $$C^{\prime}(t,q_{{{\text{seep}}}} ) \, = C(t,q_{{{\text{seep}}}} ) \, - C(t,q_{{{\text{ambient}}}} ),$$

where *θ*_ambient_ is for *θ* adjacent to the seep directions, 20°–70° and 290°–330°.

Then, *E*_*A*_ for each grid cell is adjusted, and the model run iteratively until convergence (< 1% change between iterations), generally within 5 iterations. The *E*_*A*_ adjustment applies a linear increase with distance. The linear increase with distance was based on sensitivity studies^3^. Time-varying simulations use time-centered windows, ${u\left(t,\theta \right)|}_{{t}_{1}}^{{t}_{2}}$ and ${C\left(t,\theta \right)|}_{{t}_{1}}^{{t}_{2}}$ for time *t*_1_ to *t*_2_.

### Emissions model

The emission model calculates *C′*(*t*, *x, y*) at WCS for a Gaussian plume for each grid cell above noise. Each plume has source strength, *E*_*A*_(*t*, *x*, *y*), which is initialized as *kσ*(*x*,*y*), where the initial *k* is a constant such that ∫*k*ω(*x*, *y*) is 10^5^ m^3^ day^−1^; after Hornafius et al.^6^. Thus, *k* relates ω in decibels to *E*_*A*_ in m^3^ m^−2^ s^−1^. The model also uses the WCS-measured *u*(*t*,*θ*) and typical Santa Barbara Channel marine boundary layer height, 250 m^28^.

In subsequent iterations, *k* is a function of *t* and *θ* and is calculated by comparing the simulated and observed *C′*(*t*,*θ*) at WCS. The observed *C′*(*t*,*θ*) is calculated from the angular minimum in *C*(*t*,*θ*)—around 270°—after subtraction of a Gaussian function fit to *C*(*t*,*θ*) for the northeast 330° < *θ* < 30° (Supplementary Fig. S4B). This removes terrestrial emissions from the direction of suburban communities, light industry, and commercial centers.

Each model iteration calculates a revised *k*(*t*,*θ*) from the ratio between the observed and modeled *C′*(*t*,*θ*), providing a revised *E*_*A*_(*x*,*y*) for the calculation of *C′*(*t*,*θ*) where

2 $$E_{A} \left( {x,y} \right) \, = k(t,q)s\left( {x,y} \right),$$

for each grid cell along *θ*. The model iteratively runs until convergence (0.01%), typically within 5 iterations. In addition, *k* varies linearly with distance from WCS, *r*, such that *k*(*t*,θ) = ∫*k*(*t*, *r*, θ)*dr*, a functional dependency based on *E*_*A*_ sensitivity studies with respect to *r* in Leifer et al.^22^. A linear *k*(*t*, *r*, *θ*) neither favors nearer nor further seepage. Simulations were run for three different time resolutions using WCS data averaged at the same time resolutions for each time window.

### Conversion from THC to CH_4_

*THC* from the seeps was measured in the air above Trilogy Seep at 88.5% CH_4_ with 3% ethane, 4.2% propane, and decreasing higher alkane concentrations^22^.

## Supplementary Information

Supplementary Information.

## Data Availability

WCS data 1990–2020 are published and downloadable at Leifer^29^.
