# Supplementary material for: Decadal cyclical geological atmospheric emissions for a major marine seep field, offshore Coal Oil Point, Southern California
Source: Sci Rep. 2023 Feb 21;13:3035. doi: 10.1038/s41598-023-28067-4 (PMC9944285; doi:10.1038/s41598-023-28067-4)
Supplement: Supplementary file 1 — Supplementary Information. [file 41598_2023_28067_MOESM1_ESM.pdf]

Decadal cyclical geological atmospheric emissions for a major marine seep field, offshore Coal Oil Point, Southern California

Ira Leifer
Bubbleology Research International

Supplemental Material

S.1. Seep Map

Seepage is scattered along the length of each trend, albeit non-uniformly, with strong foci in highly active, localized areas, typically at intersecting faults and associated with other geological structures such as anticlines (Leifer et al., 2010), creating focused seep areas. The largest of these focused seep areas have been informally named (**Table S1**).

Table S1. Location and direction from West Campus Station of informally-named seeps.

Seep Area	q	Latitude, Longitude
La Goleta Seep	152°	34° 23.503'N, 119° 51.193'W
Seep Tent Seep	198°	34° 23.063'N, 119° 53.428'W
Platform Holly	238°	
Trilogy Seep C	178°	34° 23.634'N, 119° 52.702'W
Trilogy Seep B	178°	34° 23.620'N, 119° 52.709'W
Trilogy Seep A	178°	34° 23.603'N, 119° 52.699'W
Patch Seep	140°	34° 21.850'N, 119° 49.755'W
Shane Seep	230°	34° 24.370'N, 119° 53.428'W
IV Super Seep	146°	34° 24.090'N, 119° 52.066'W
Tonya Seep	184°	34° 24.043'N, 119° 52.841'W
Horseshoe Seep	186°	34° 23.799'N, 119° 52.519'W
Rostocker Seep	99°	34° 24.230'N, 119° 50.438'W
Seadog Seep	240°	34° 24.172'N, 119° 51.212'W

*θ– direction from West Campus Station (34.414949°N, 119.879690°W).

S.2. West Campus Station Time Series Data

Significant variations on daily to seasonal to interannual time scales are apparent in the WCS time series (**Fig. S1**). Concentrations and emissions hereafter are for total hydrocarbon, *THC*. WCS data quality improved significantly in 2008, with measurements decreasing from 1-hour to 1-minute time resolution and an extended measurement range that allowed higher values of C and u to be recorded.

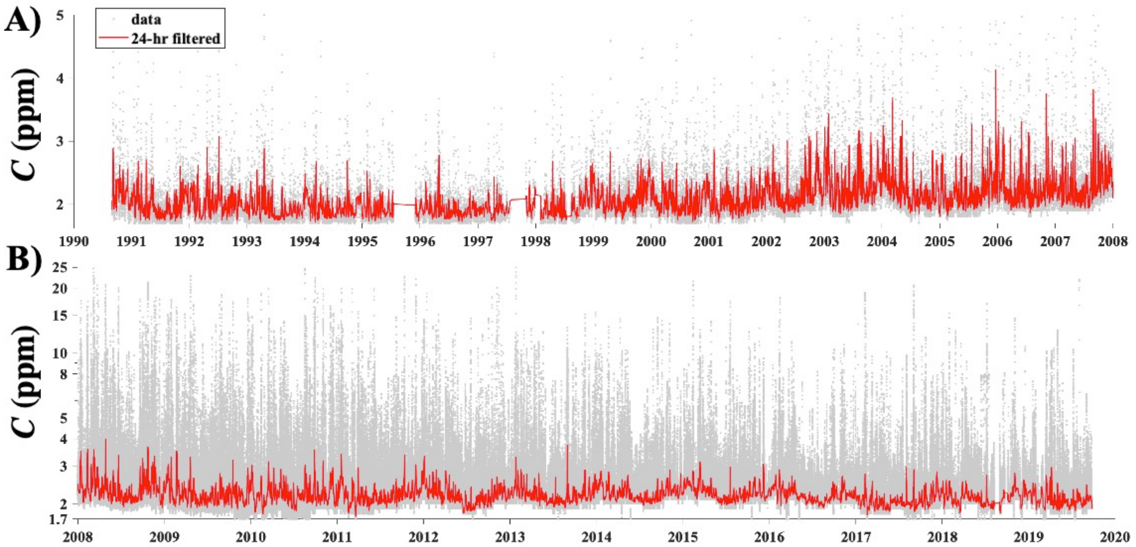

Fig. S1. Concentration, *C*, for seep directions (90<θ<270°) and 1-day averaged for **A)**1990-2007, **B)** 2008-2020. Note different scales on each panel. Data key on figure.

S.3. Seep Trends

$C(t, \theta_{Seep})$ for WCS decreased slowly from 1990 to 1995 and then generally increased through 2008 (with short maxima and minima in 2003 and 2005), followed by a decline through 2017 (**Figs. 4, S4**). The seasonal trend was best fit by a least-squares linear regression by a two-part 365.3-day period (124-ppb amplitude) sine function with a peak in winter. The discrete fit accounted for a small phase offset around 2008. The sinusoidal function was subtracted from the 2008-2020 data and then fit with an exponential function by a least-squares linear-regression analysis (**Fig. S2**). The seasonal trend likely relates partly to wind speed (which induces waves), particularly associated with storms.

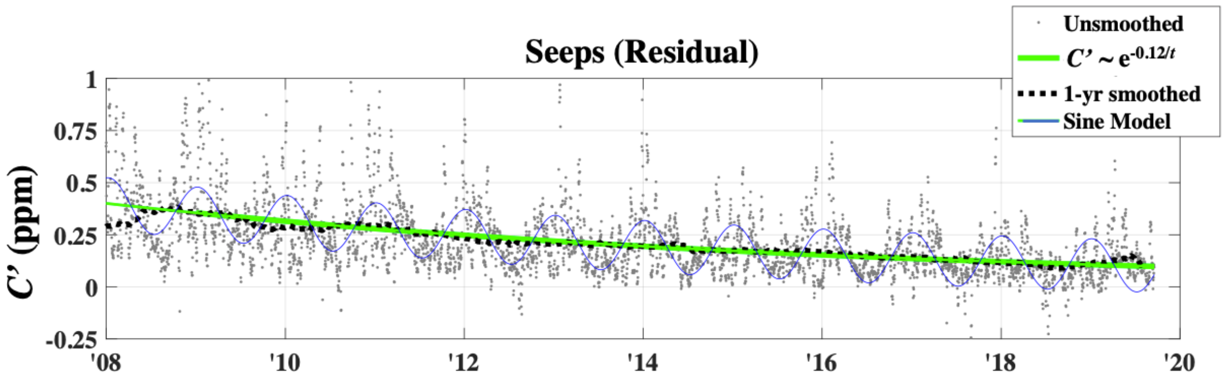

Fig. S2. Anomaly total hydrocarbon concentration, *C'*, and 1-year running-averaged smoothed *C* for 2008-2020 for seep directions (90-270°), single sinusoidal function fit, and exponential fit. Data key on figure.

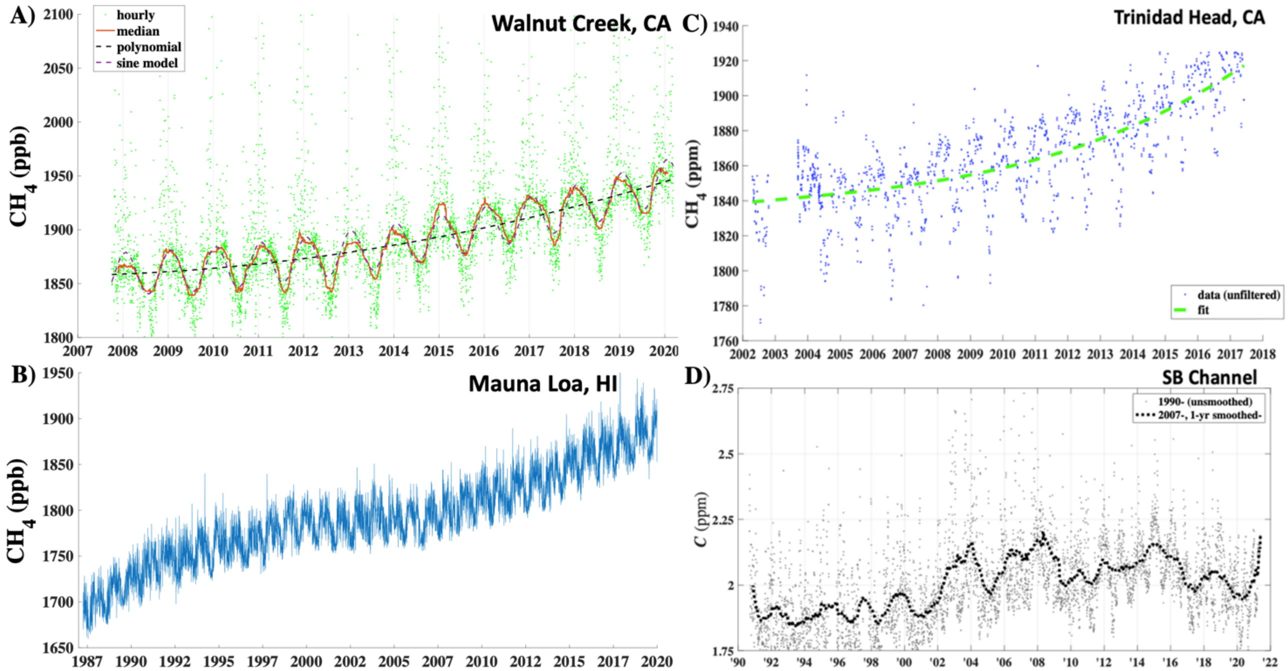

Fig. S3. A) Walnut Grove CH_4 data, 2008-2020, median filtered, least-squares linear-regression polynomial fit, and sinusoidal fit. **B)** Mauna Loa CH_4 data 1987-2020. **C)** Trinidad Head CH_4 2002-2017 and least-squares linear-regression polynomial fit. Station data from NOAA (2021). **D)** Santa Barbara Channel (WCS) non-seep direction trend (**Fig. 4B**).

S.4. California and Northern Hemisphere Methane Trends

The overall WCS $C(t, \theta_{\text{seep}})$ trend differs dramatically from the California CH_4 trend, represented by the Walnut Grove tall tower, and the CH_4 trend for the Northern Hemisphere, represented by Mauna Loa, Hawaii (**Fig. S3**). Hourly CH_4 concentrations for the Walnut Grove tall tower (inlet at 423 m) were rolling-median filtered (75 hours) to remove short excursion from local sources, leaving a seasonal cycle superimposed on interannual growth. The interannual trend was fit with a 2nd-order polynomial (R^2=0.73) with an average increase of 7.28 ppb yr^{-1}. A single sinusoid was well fit to the detrended residual with a 1-yr cycle, peaked in winter, which found an amplitude of 20.2 ppb (R^2=0.8). The winter peak large arises from the lower planetary boundary layer as many emission sources are at a minimum in winter. The California cycle runs counter to the seasonality of most emission sources (microbial from wetlands and agricultural emissions are temperature sensitive and thus higher in the summer) and results from a shallower winter boundary layer than in the summer (Mark Fischer, personal communication, 2020). Seasonal changes in hydroxyl concentration and resultant CH_4 loss also play a role.

Mauna Loa, Hawaii CH_4 data showed rapid growth in the late 1980s, stabilizing from ~2000 to 2008, before growing approximately linearly at 7.54 ppb yr^{-1}. The seasonal cycle in the Mauna Loa data was 11 ppb from a sinusoidal fit to the residual of a 3rd-order polynomial fit. Mauna Loa seasonality is much less than the seep field background seasonality.

In part, this is due to the higher altitude of Mauna Loa (and Walnut Creek); however, Trinidad Head is at sea level and also exhibits a much smaller seasonal cycle than the seep field. Notably, the Seep Field background trend is very different from the NOAA station trends and is at much higher concentrations. This difference demonstrates the importance of local seep field emissions in the

Santa Barbara basin, where recirculation of CH_4-laden waters occurs due to gyre-like currents (Leifer, 2019) and air that can recirculate as part of the sea-breeze cycle (Dorman and Winant, 2000). Additionally, the largest or second largest in terms of oil emissions seep field in California, is situated to the west of the COP seep field (prevailing upwind), the extensive Concepcion Seep Field, where gas seep bubbles have been documented (Leifer, 2019). Currently, no emissions assessments are available. This strongly suggests that seepage has a significant impact on Santa Barbara Channel CH_4, with contributions from other regional sources.

S.5. Direction Resolved Seep Trends

Prevailing winds are strongest from the west due to topographic forcing from the coastal mountain range – in this direction, concentrations are the lowest, which is consistent with no mapped seepage in that direction (**Fig. S4**).

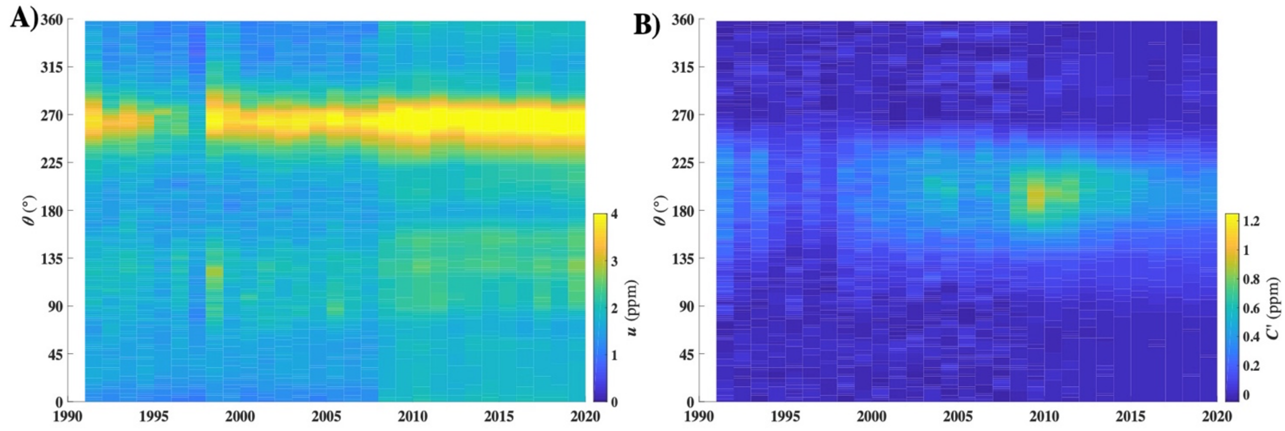

Fig. S4. A) Wind speed, u, versus wind direction, θ, and time, t. **B)** Concentration anomaly, $C'(t,\theta)$, versus θ and t after subtraction of Gaussian function fit to the northeast plume. Calculated for 1-year time windows.

S.6. Modeled Emissions

The Cycle simulation (**Fig. S5**) was well fit by an offset sinusoidal function with amplitude (94,000 m^3 day^{-1}), and a 26.3-year period around a cycle average of 90,300 m^3 day^{-1}). The exponential decrease for the Annual simulation (**Fig. 8A**) had a 10.2-year time scale; however, the seasonal simulation (**Fig. S6**) found a shorter time scale – 9.6 years.

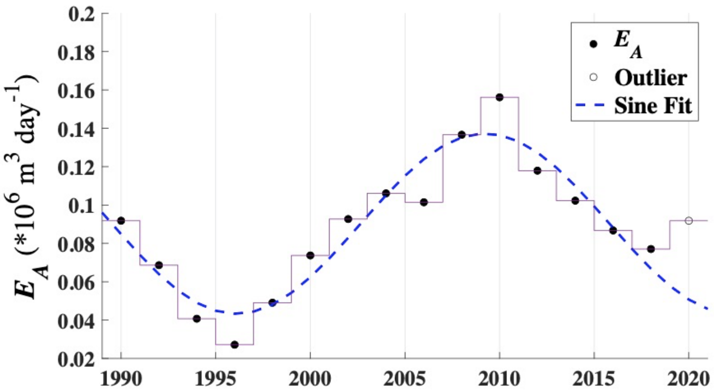

Fig. S5. COP seep field atmospheric emissions, E_A, Cycle simulation, and sinusoidal fit by least-squares linear-regression analysis.

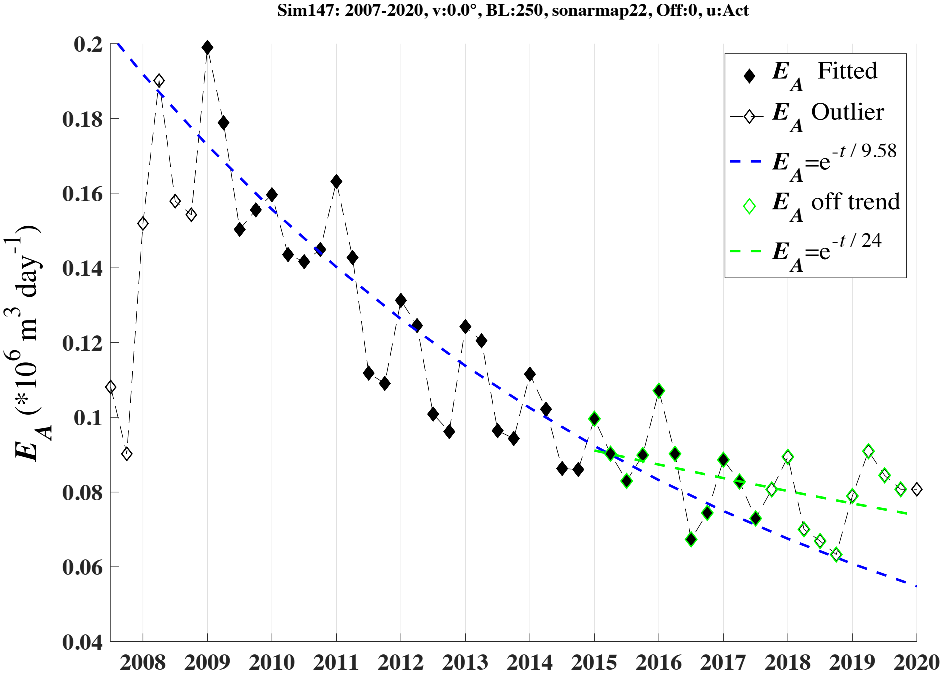

Fig. S6. COP seep field atmospheric emissions, E_A, Seasonal simulation, and least-squares linear-regression analysis exponential curve fit.

Individual time-window simulation maps (**Figs. S7-S8**) show the same overall trends as *C'* (**Fig. 4**), with an E_A minimum in 1995 and a peak around 2009-2010. Using the higher quality data since 2008, 1-year simulations showed a peak E_A of 156,000 m^3 day^{-1}. This was approximately six times the minimum of 27,200 m^3 day^{-1}.

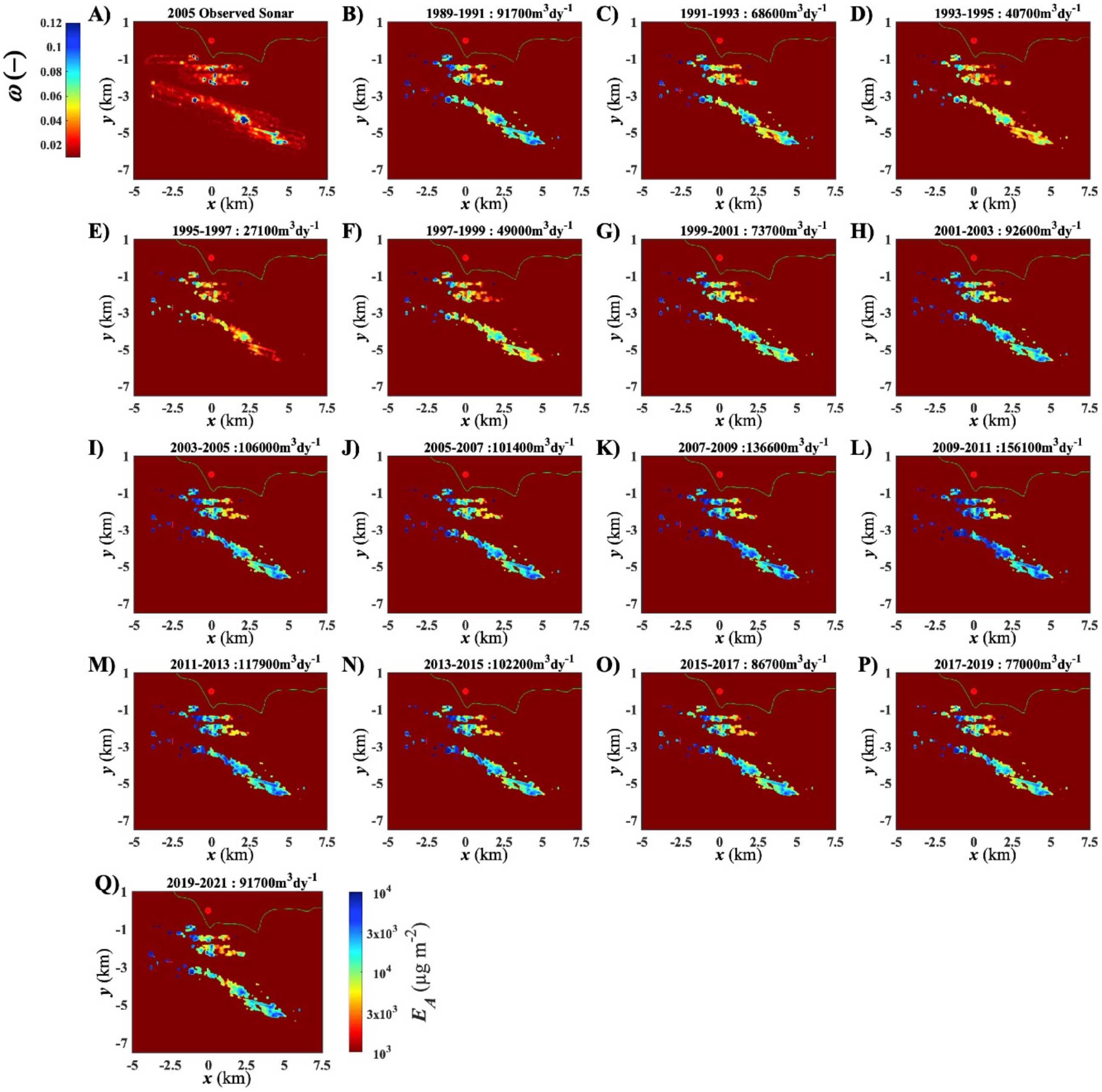

Fig. S7. A) COP seep field 2005 sonar return, ω, 22/56-m gridding, **b-p)** Atmospheric emissions, E_A, 2-year averages every 2 years, 1990-2020, time periods labeled on panels. The red dot shows WCS location and coordinate system origin; the green line shows the coastline.

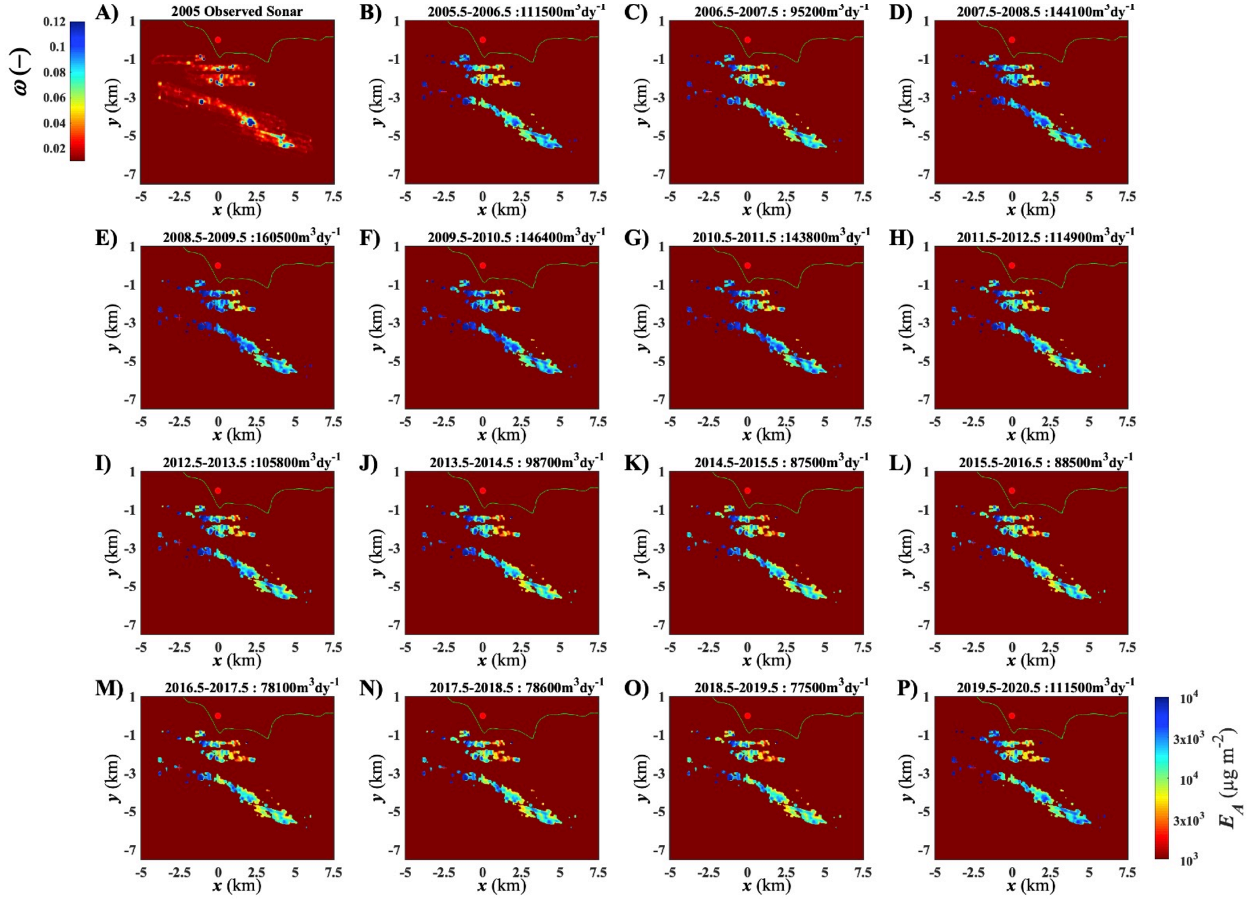

Fig. S8. A) COP seep field 2005 sonar return, ω, 22/56-m gridding, same as **Fig. 2**, **B-M)** Atmospheric emissions, E_A, 1-year averages – Annual simulation, 2006-2020, time periods labeled on panels. The red dot shows WCS location and coordinate system origin; the green line shows the coastline.

S.7. Methodological Differences

The primary difference between a sonar survey seep field emission estimate and an air quality station emissions estimate is that the latter is continuous, including the stormy season when sonar surveys cannot be safely conducted. In contrast, sonar surveys only provide a snapshot of a phenomenon that is highly variable, see review in Leifer (2019). The simulations show a clear seasonal trend (**Fig. S6**), with a summer minimum when sonar surveys are preferentially scheduled due to calmer winds (**Fig. 2A**) and lower waves. Also, during summer, large transient emissions are at a minimum. Seasonality was not addressed by either sonar survey. In contrast, WCS data are continuous.

Other sources of variability include tidal variations (Boles et al., 2001), diurnal wind cycles (Leifer et al., 2021), and transient seepage eruptions, which are challenging to address in a sonar survey. Furthermore, sonar surveys are spatially limited, although emissions can (and do) occur from areas outside the survey area – areas that may be active in other seasons. In this regard, Padilla et al. (2019) significantly under-surveyed the seep field (4.1 of 18 km^2).

By contrast, the plume inversion approach captures emissions, including variations and transient emissions and emissions from outside the surveyed area of the seep field. As such, the near

cessation of emissions during the seep field minima, which is when Hornafius et al. (1999) surveyed the field decreased the time-period averaged E_A derived by the model.

Another potential bias in Hornafius et al. (1999) was the sonar calibration by air bubbles from a compressed tank on the seabed. This calibration did not address the faster dissolution rates for seep methane bubbles versus air bubbles. Leifer et al. (2017) showed E_B overestimated by 25% for a sonar window at 15 m above the seabed. This effect on E_B likely was larger as the Hornafius et al. (1999) sonar window was 30-40 m above the seabed and contributed to increasing E_B from Hornafius et al. (1999).

The Padilla et al. (2019) survey found E_B =24,000 m^3 day^{-1}, significantly less than the model-estimated E_B of 165,000 m^3 day^{-1}. A significant contribution to this discrepancy is the small survey coverage of only 4.1 km^2, a quarter of the survey area of Hornafius et al. (1999). Additionally, the early fall emissions from Padilla et al. (2019) miss the far stronger winter emissions and large transient events, which also tend to occur in winter.

Another consideration is that the sea:air partitioning could be greater than 50% in the water column. This partition depends on several factors, with larger bubbles, oilier bubbles, and stronger bubble plumes decreasing the water component of the partitioning (Leifer et al., 2006; Leifer and Patro, 2002). Note, upper water-column bubble dissolution contributes to E_A. For strong plumes, the dissolved partition is small based on the similarity between atmospheric and seabed CO_2 concentration – i.e.; upwelling transport is highly efficient. Clark et al. (2000) assessed the water column fraction for the entire field, i.e., combining the effect of larger and smaller plumes for a 1215 Aug. 1996 survey, i.e., roughly contemporaneous with Hornafius et al. (1999).

Supplemental References

Boles JR, Clark JF, Leifer I, Washburn L. 2001. Temporal variation in natural methane seep rate due to tides, Coal Oil Point area, California. *Journal Geophysical Research - Oceans* **106**(C11): 27,077-27,086. doi:10.1029/2000JC000774.

Clark JF, Washburn L, Hornafius JS, Luyendyk BP. 2000. Dissolved hydrocarbon flux from natural marine seeps to the southern California Bight. *Journal of Geophysical Research - Oceans* **105**(C5): 11509-11522. doi:10.1029/2000JC000259.

Dorman CE, Winant CD. 2000. The structure and variability of the marine atmosphere around the Santa Barbara Channel. *Monthly Weather Review* **128**(2): 261-282. doi:10.1175/1520-0493(2000)128<0261:TSAVOT>2.0.CO;2.

Hornafius SJ, Quigley DC, Luyendyk BP. 1999. The world's most spectacular marine hydrocarbons seeps (Coal Oil Point, Santa Barbara Channel, California): Quantification of emissions. *Journal Geophysical Research - Oceans* **104**(C9): 20,703-20,711. doi:10.1029/1999JC900148.

Leifer I. 2019. A synthesis review of emissions and fates for the Coal Oil Point marine hydrocarbon seep field and California marine seepage. *Geofluids* **2019**(4724587): 1-48. doi:10.1155/2019/4724587.

Leifer I, Chernykh D, Shakhova N, Semiletov I. 2017. Sonar gas flux estimation by bubble insonification: Application to methane bubble flux from seep areas in the outer Laptev Sea. *The Cryosphere* **11**(3): 1333-1350. doi:10.5194/tc-11-1333-2017.

Leifer I, Kamerling M, Luyendyk BP, Wilson D. 2010. Geologic control of natural marine hydrocarbon seep emissions, Coal Oil Point seep field, California. *Geo-Marine Letters* **30**(3-4): 331-338. doi:10.1007/s00367-010-0188-9.

Leifer I, Luyendyk BP, Boles J, Clark JF. 2006. Natural marine seepage blowout: Contribution to atmospheric methane. *Global Biogeochemical Cycles* **20**(3): GB3008. doi:10.1029/2005GB002668.

Leifer I, Melton C, Blake DR. 2021. Long-term atmospheric emissions for the Coal Oil Point natural marine hydrocarbon seep field, offshore California. *Atmospheric Chemistry Physics* **21**(23): 17607–17629. doi:10.5194/acp-21-17607-2021.

Leifer I, Patro R. 2002. The bubble mechanism for methane transport from the shallow seabed to the surface: A review and sensitivity study. *Continental Shelf Research* **22**(16): 2409-2428. doi:10.1016/S0278-4343(02)00065-1.

NOAA. 2021. Trends in Atmospheric Methane - Earth System Research Laboratories. NOAA.

Padilla AM, Loranger S, Kinnaman FS, Valentine DL, Weber TC. 2019. Modern assessment of natural hydrocarbon gas flux at the Coal Oil Point seep field, Santa Barbara, California. *Journal of Geophysical Research: Oceans* **124**(4): 2472-2484. doi:10.1029/2018jc014573.
